# Genotyping and phylogeography of infectious bronchitis virus isolates from Pakistan show unique linkage to GI-24 lineage

**DOI:** 10.1016/j.psj.2023.103236

**Published:** 2023-10-24

**Authors:** Waqar Saleem, Nick Vereecke, Muhammad Goher Zaman, Farhan Afzal, Iqra Reman, Saeed ul-Hasan Khan, Hans Nauwynck

**Affiliations:** ⁎ Laboratory of Virology, Department of Translational Physiology, Infectiology and Public Health, Faculty of Veterinary Medicine, Ghent University, 9820 Merelbeke, Belgium; † Pathosense BV, Lier 2500, Belgium; ‡ Department of Zoology, Faculty of Biological Sciences, Quaid-i-Azam University, 54770 Islamabad, Pakistan; § Disease Diagnostic Laboratory, Poultry Research Institute, 46000 Rawalpindi, Pakistan

**Keywords:** BEAST, poultry, infectious bronchitis, viral evolution, Pakistan

## Abstract

Infectious bronchitis virus (**IBV**) is prevalent in Pakistan causing enormous economic losses. To date no clear data are available on circulating genotypes and phylogeographic spread of the virus. Hence current study assessed these parameters for all available IBV Pakistani isolates, based on the 9 new sequences, with respect to other Asian and non-Asian countries. Results indicated that all Pakistani isolates belonged to genotype I (**GI**), with more than half of them (16/27) belonging to the GI-24 lineage, against which no vaccine is available. Three possible introduction events of the GI-13 IBV lineage into Pakistan, based on the estimated IBV population using isolates from this study, were observed possibly from Afghanistan, China, and/or Egypt. These events were further analyzed on the S1 amino acid level which showed unique alterations (S250H, T270K, and Q298S) in 1 isolate (IBV4, GI-13) when compared to GI-1 lineage. Both GI-1 and GI-13 Pakistani strains showed close homology with homologous vaccine strains that are used in Pakistan. For GI-24 strains, none of the used vaccines showed substantial homology, necessitating the need for further exploration of this lineage and vaccine design. In addition, our findings highlight the importance of genomic surveillance to support phylogeographical studies on IBV in genotyping and molecular epidemiology.

## INTRODUCTION

Infectious bronchitis (**IB**) is one of the most important infectious diseases of poultry industry due to its contagious nature and significant economic losses (Umar et al., 2018). It is caused by the infectious bronchitis virus (**IBV**), which is part of the *Gammacoronavirus* genus and *Coronaviridae* family. This group of viruses is featured by a positive-sense RNA genome of about 27.6 kb in size (Lin and Chen, 2017). Transmission of the virus mainly occurs through aerosols, direct contact, and fomites, with evidence of vertical transmission (Pereira et al., 2016). The disease manifests itself in many clinical forms and affects a variety of organs including respiratory (Cavanagh et al., 2005), urinary (Boroomand et al., 2012), and reproductive systems (Sylvester et al., 2005). Productivity of the birds is severely affected due to production of misshapen eggs and decreased laying rate leading to economic losses (Dave, 2007). Problems with IB are seen worldwide with various outbreak reports from the United States (Toro, 2010), Latin America (Villarreal et al., 2010), Europe (Capua et al., 1999; Herdt et al., 2016), Asia (Feng et al., 2017), and Africa (Roussan et al., 2008) over the last years. Also in Pakistan, IBV is prevalent as suggested by data from seroprevalence studies from 2004 (Mahmood et al., 2004), 2005 (Hussain et al., 2005), and 2007 (Ahmed et al., 2007). Even though vaccination is done on routine basis against IB, failure is often observed due to continuous emergence of new strains (Huang et al., 2004).

The IBV genome comprises 2 untranslated regions (**UTRs**) at the 5′ and 3′ ends, along with 2 overlapping open reading frames (**ORFs**), encoding for the polyproteins 1a and 1ab (Valastro et al., 2016). In addition, the IBV virion harbors structural proteins consisting of Spike (**S**), envelop (**E**), matrix (**M**), and nucleocapsid (**N**) proteins. Also, 2 accessory genes, ORF3 and ORF5, resulting in proteins 3a and 3b and 5a and 5b, respectively, have also been described (Hodgson et al., 2006). The S protein is responsible for its pathogenicity and immunogenicity as it is involved in the virus-cell interaction, which is followed by its post-translational modification into 2 subunits, called S1 and S2 (Dave, 2007). The S1 subunit is mainly responsible for antigenic variation of IBV due to its 3 hypervariable regions (**HVRs**) (Saadat et al., 2017). This is majorly due to evolutionary forces like genetic drifts and recombination, adaptation to host cell receptors and immune evasion (Moya et al., 2004). This is facilitated in part by the presence of an RNA-dependent RNA Polymerase (**RDRP**) which lacks proof-reading activity (Duffy et al., 2008). Hence, antigenic determinants of the S1 protein are changed leading to development of new serotypes and genotypes (Promkuntod et al., 2015). In general, the S1 glycoprotein of IBV serotypes varies by approximately 20 to 25%. However, a difference up to 50% has been observed, which affects the cross-protection against newly emerging or re-emerging virus strains (Ennaji et al., 2020), making control of disease through vaccination very difficult (Cavanagh et al., 2005; Nabih et al., 2018). More than 50 genetically and antigenically different types of IBV have been identified worldwide (Capua et al., 1999; Valastro et al., 2016) and circulation of M-41 and 4/91 (793/B serotype) strains has been reported in Pakistan (Ahmed et al., 2007). Based on the full S1 sequence, a new system for IBV genotype classification is also suggested which categorizes IBV in 7 genotypes (GI–VII) with multiple lineages (Valastro et al., 2016; Houta et al., 2021). Commonly circulating Pakistani strains like M-41 belongs to the GI-1 lineage, while 4/91 belong to GI-13 (Rohaim et al., 2019; Sheng et al., 2020). The emergence of these new genotypic lineages and strains also vary in their pathogenicity and target organs making IB a multisystemic disease (Meulemans et al., 2001).

Due to its genetic variety, the S1 gene of IBV is most often targeted in molecular epidemiological studies to characterize (e.g., genotyping) and understand the spread of the virus (Bande et al., 2017). Reverse transcriptase PCR (**RT-PCR**) and quantitative reverse transcriptase PCR techniques have been developed in last 3 decades for this purpose (Acevedo et al., 2013). Through sequencing and phylogenomics, the route of virus distribution and identification of strains prevalent in a particular area can be established (Zulperi et al., 2009). In a developing country like Pakistan, studies on the molecular epidemiology of local IBV isolates are limited. One of these studies classified a Pakistani IBV isolate in the GI-13 lineage that includes both the vaccine and virulent field strains, previously assigned to the 4/91 (793/B serotype) like (Rafique et al., 2018a). Another study found an 82 to 93% nucleotide homology of a Pakistani isolate with Indian isolates (Rafique et al., 2018b). Nevertheless, these studies often lack substantial information on isolation date, location, and sample origin (e.g., tissue), which empower and drive accuracy of phylodynamic. Still, this type of analysis on local IBV samples is important due to a consistent prevalence of the disease in Pakistan and its multisystemic pathogenic nature.

This study is the first to deliver data on the phylogeography of all the reported IBV isolates in Pakistan. Here, existing IBV sequences from Pakistan, neighboring Asian, and some non-Asian countries were supplemented with 9 new Pakistani sequences from IBV field isolates. These were used for genotyping and a Bayesian Evolutionary Analysis Sampling Trees (**BEAST**) analysis to assess evolutionary rate, phylogenetic relatedness, and map potential introduction events into Pakistan. Isolation source of the isolates were also analyzed to map possible links between different circulating genotypes.

## MATERIALS AND METHODS

### Sampling and RNA Extraction for New IBV Field Isolates

Tissue samples from trachea/lungs (*n* = 18) and oviduct (*n* = 7) from 25 suspected cases of IBV were taken during a disease outbreak period between October 2017 to February 2018 from various districts of Punjab. PureLink RNA Mini Kit (Invitrogen, Cat No. 12183025) along with TRIzol Reagent (Invitrogen, Cat No. 15596026) was used for RNA extraction according to company guidelines. Extracted RNA was stored at −80°C.

### Reverse Transcription, Nested RT-PCR, and Sequencing

RevertAid First Strand cDNA Synthesis Kit (Thermo Scientific, Cat No. K1621) was used for reverse transcription with gene-specific primer (SX2−) according to the company's guidelines. Briefly, incubation was done at 42°C for 60 min, 25°C for 10 min and again at 42°C for 2 h. Finally, mixture was incubated at 70°C for 10 min to inhibit reverse transcriptase. cDNA was stored at −20°C.

RT-PCR primers and conditions were used as previously described for partial amplification of S1 gene (Cavanagh et al., 1999; Jones et al., 2005; Worthington et al., 2008). The sequence of the primers is shown in Table 1. For the first PCR reaction, SX1+ and SX2− (10 nmol each); 2.5 μL of 10× PCR buffer (100 mM Tris-HCl, pH 9.0, 500 mM KCl, 1% Triton X-100); 1.75 μL of 25 mM MgCl_2_; 0.5 μL of 10 mM dNTPs; 2.5 U DreamTaq DNA Polymerase (Thermo Scientific, Cat No. EP0702) and nuclease-free water to make final reaction volume 25 μL. For nested PCR, 8 μL of first PCR product was used with SX3+ and SX4− primers; the rest of the ingredients were same. Nuclease-free water was adjusted to make final reaction volume 25 μL. The PCRs were performed using the following conditions: denaturation (94°C, 1 min), annealing (48°C for 1.5 min), extension (72°C, 2 min) for a total of 30 cycles. The expected 393 bp product was purified and used for sequencing.

**Table 1:** Primers used for nested PCR.

Name	Sequence	Position on S1 gene
X1+	5′-CACCTAGAGGTTTGYTWGCAT-3′	677–698
X2−	5′-TCCACCTCTATAAACACCYTT-3′	1148–1168
X3+	5′-TAATACTGGYAATTTTTCAGA-3′	705–725
X4−	5′-AATACAGATTGCTTACAACCACC-3′	1075–1097

Purified cDNA samples were sent to Macrogen, Inc. (South Korea) for sequencing along with internal primers SX3+ and SX4−. Sequencing results were used to generate a consensus sequence by Geneious v10.2.5 on which 2 sets of primers were designed (Table 2) for further sequencing to omit errors.

**Table 2:** Names and sequences of new primers designed for sequencing.

Name	Sequence	Position on consensus sequence
BV-310F	5′-ACTGTGTCACTTGCTTACGGA-3′	310–330
BV-246F	5′-AACAAATACTGCTCAGGATGGT-3′	246–267
BV-235R	5′-GGAAAGTATCAACGCCACCTG-3′	235–214
BV-309R	5′-ATACACAAAACTACTCAGAAA-3′	309–289

### Genotyping

All sequences were used in a phylogenetic analysis to allow genotyping based on previously published genotyping schemes (Valastro et al., 2016; Fan et al., 2019; Rohaim et al., 2020; Ali et al., 2022). All sequences were combined into a single FASTA file and aligned using MAFFT (v.7.453; Katoh and Standley, 2013). Extra sequences at 5′ and 3′ ends were removed to align with the new S1 amplicons. The resulting multiple sequence alignment file was used to infer phylogenetic relationship using IQtree2 (v.2.2.0; Minh et al., 2020) with 1,000 ultrafast bootstraps (-bb). The treefile was visualized and annotated in iTOL (v.5; Letunic and Bork, 2021) to address genotypes. An overview of sequences by countries and sample types can be found in Supplementary Table 1 and list of NCBI accession numbers in Supplementary Table 2.

### Phylogeographic Analysis

The obtained sequences were supplemented with IBV sequences available from the NCBI database, including all Pakistani sequences (*n* = 27) and sequences from other Asian countries (*n* = 77). Also, some non-Asian isolates (*n* = 16) were included in the analysis. A multiple sequence alignment was performed using MAFFT (v.7.453; Katoh and Standley, 2013) after which extra sequences at 5′ and 3′ ends were removed to align with the new S1 amplicons. To remove putative recombination events from the data, an RDP5 (v.5; Martin et al., 2020) was performed at *default* settings. Sequences showing support for recombination in a minimum of 3 methods were removed. An initial maximum-likelihood (**ML**) tree was constructed using IQtree2 (v.2.2.0; Minh et al., 2020) with 1,000 ultrafast bootstraps (-bb) to assess the temporal signal in the resulting data in Tempest (v.1.5.3; Rambaut et al., 2016). After which 1 IBV strain (KU686875) was considered an outlier and was removed from further analyses. Next, a BEAST (v.1.10.4; Suchard et al., 2018) analysis was initiated under the TN93+G4 model (Yang et al., 2013; Franzo et al., 2020). To perform phylogeographic analyses, country information was included as discrete trait under a symmetric substitution model (including to infer social network with BSSVS). The molecular clock was set at an uncorrelated relaxed clock (lognormal distribution) and strict clock for the sequences and countries, respectively. The analysis was run under a Bayesian SkyGrid coalescent and the population size was estimated with 100 parameters and random starting tree. Five independent runs of 50,000,000 chains, with tree sampling after 5,000 steps, were run on the HPC Tier2 cluster Yoltik (GPU) at Ghent University. Resulting tree and logfiles from independent runs were combined using logcombiner (v.1.10.4; Suchard et al., 2018) and final trees were annotated in treeannotator (v.1.10.4; Suchard et al., 2018), prior to visualization in Figtree (v1.4.4; Rambaut, 2018). Convergence (a minimum of 150 for each parameter) of all effective sample size (**ESS**) measures was observed in Tracer (v.1.7.2; Rambaut et al., 2018).

### Protein S1 Subunit Analyses

All Pakistani sequences were supplemented with vaccine strains that are used in Pakistan (GI-1: H120 (KF188436), M41 (X04722), and H52 (AF352315); GI-13: 4/91 (KF377577); GI-17: KM91 (FJ807946)) and other main GI vaccine strains as described by Guzmán and Hidalgo (Guzmán and Hidalgo, 2020) (GI-1: Ma5 (AY561713) and Connecticut (KF696629); GI-5: Armidale (KU556805); GI-6: VicS (KF460437); GI-12: D274 (MH021175); GI-13: CR88121 (JN542567) and 1/96 (MK680010); GI-19: Arkansas (GQ504721); GI-23: 1212B (KU979007)). For vaccine strains B48, 249G, and PL84084 no publicly available sequence could be found. These were used in a new multiple sequence alignment using MAFFT (v.7.453; Katoh and Standley, 2013). These sequences were further analyzed in MEGA (v.11; Tamura et al., 2021) to translate nucleotide sequences into protein sequences.

## RESULTS

### RT-PCR Results

**Figure 1:**
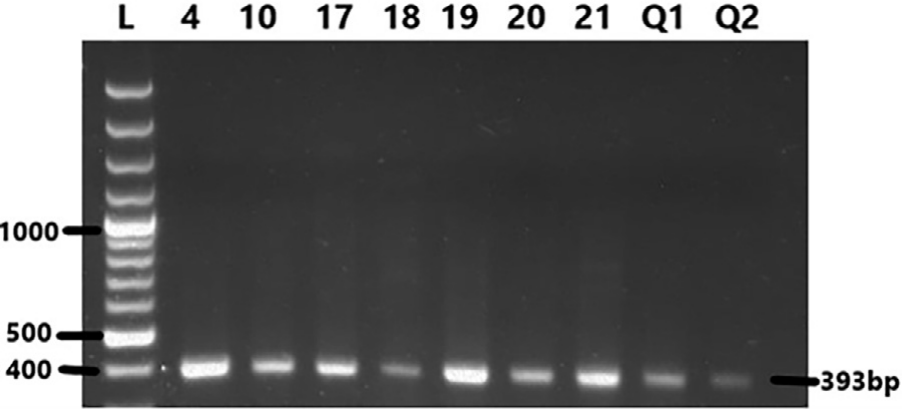
RT-PCR gel picture showing the 393 bp band for 9 IBV isolates used in this study.

Nine samples (trachea/lungs = 7; oviduct = 2) were declared positive by nested RT-PCR and were used for sequencing showing a 393 bp band (Figure 1). These isolates were sequenced and submitted to NCBI's GenBank under accession numbers MH703655-MH703663.

### IBV Genotyping and Link With Sampling Origin

**Figure 2:**
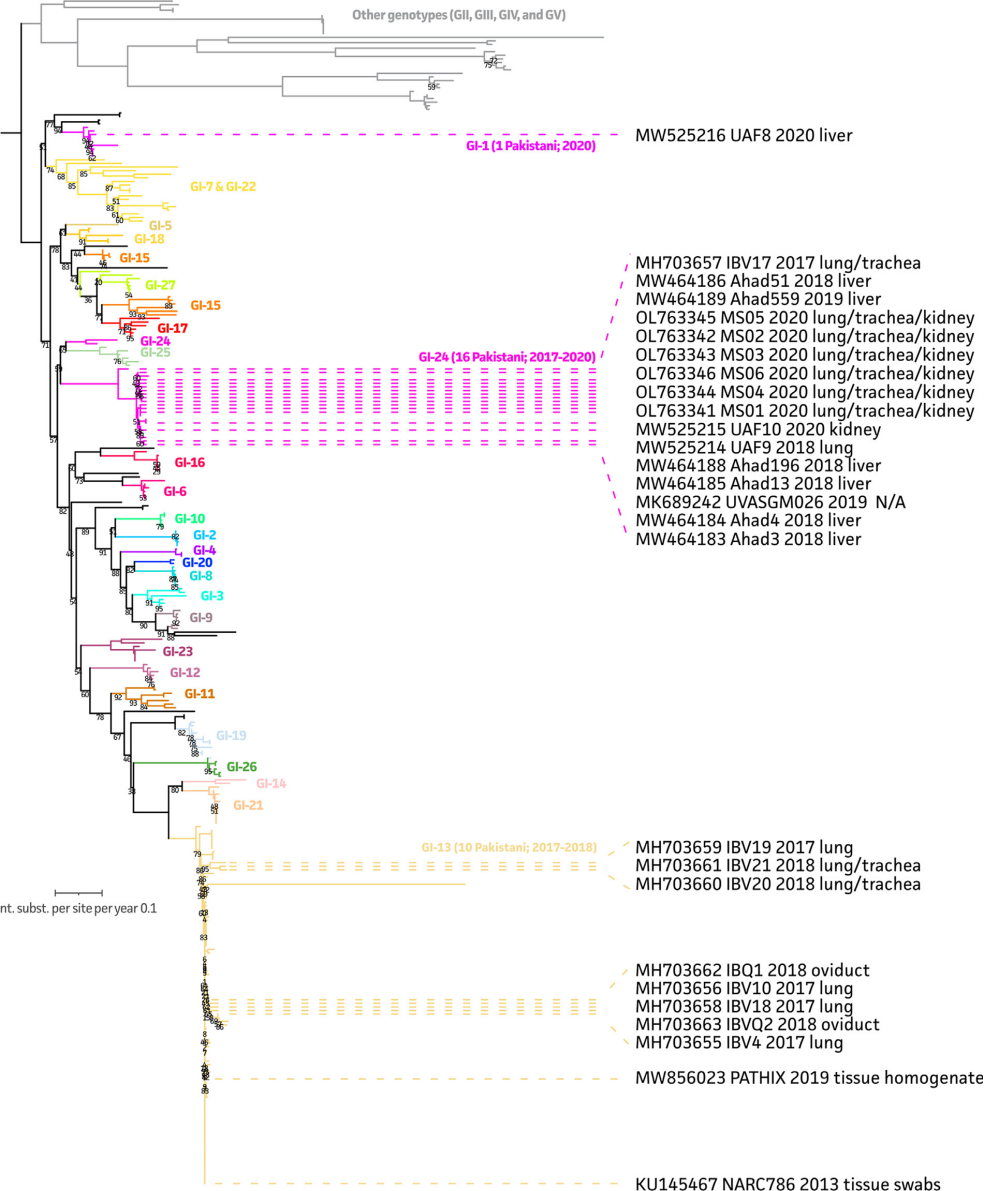
Maximum likelihood (ML) tree of Pakistani IBV S1 sequences and a selection of different genotypes. The ML tree represents most variety of genotypes that are known within the IBV species by the inclusion of all available Pakistani sequences (18 existing and 9 new ones from this study) along with sequences from various genotyping manuscripts (Fan et al., 2019; Rohaim et al., 2020; Ali et al., 2022). Genotypes II, III, IV, and V are indicated in gray at the top of the ML tree. The genotype I strains are subdivided into different lineages as reported by Ali et al. (2022). Only Pakistani sequences were annotated by highlighting the dotted extended branches in brown.

Our 9 new sequences were subjected to genotyping, using a reduced phylogenetic inference, including most relevant genotypes. As shown in Figure 2, all isolates belonged to genotype 1 (GI). Previously sequenced isolates from Pakistan, as available from NCBI, were classified into 2 different lineages within GI. Most of these strains (*n* = 16) belonged to the GI-24 lineage. Interestingly, 1 isolate (UAF-8; MW525216) was shown to be a GI-1 lineage strain. Among our 9 new sequences, 1 belonged to the GI-24 lineage (IBV17, MH703657), while the other 8 belonged to the GI-13 lineage. GI-13 lineage strains are associated with samples primarily isolated from the respiratory and reproductive tracts while GI-24 lineage includes isolates from liver and kidney in addition to the respiratory tract.

### Introductions of IBV Into Pakistan

**Figure 3:**
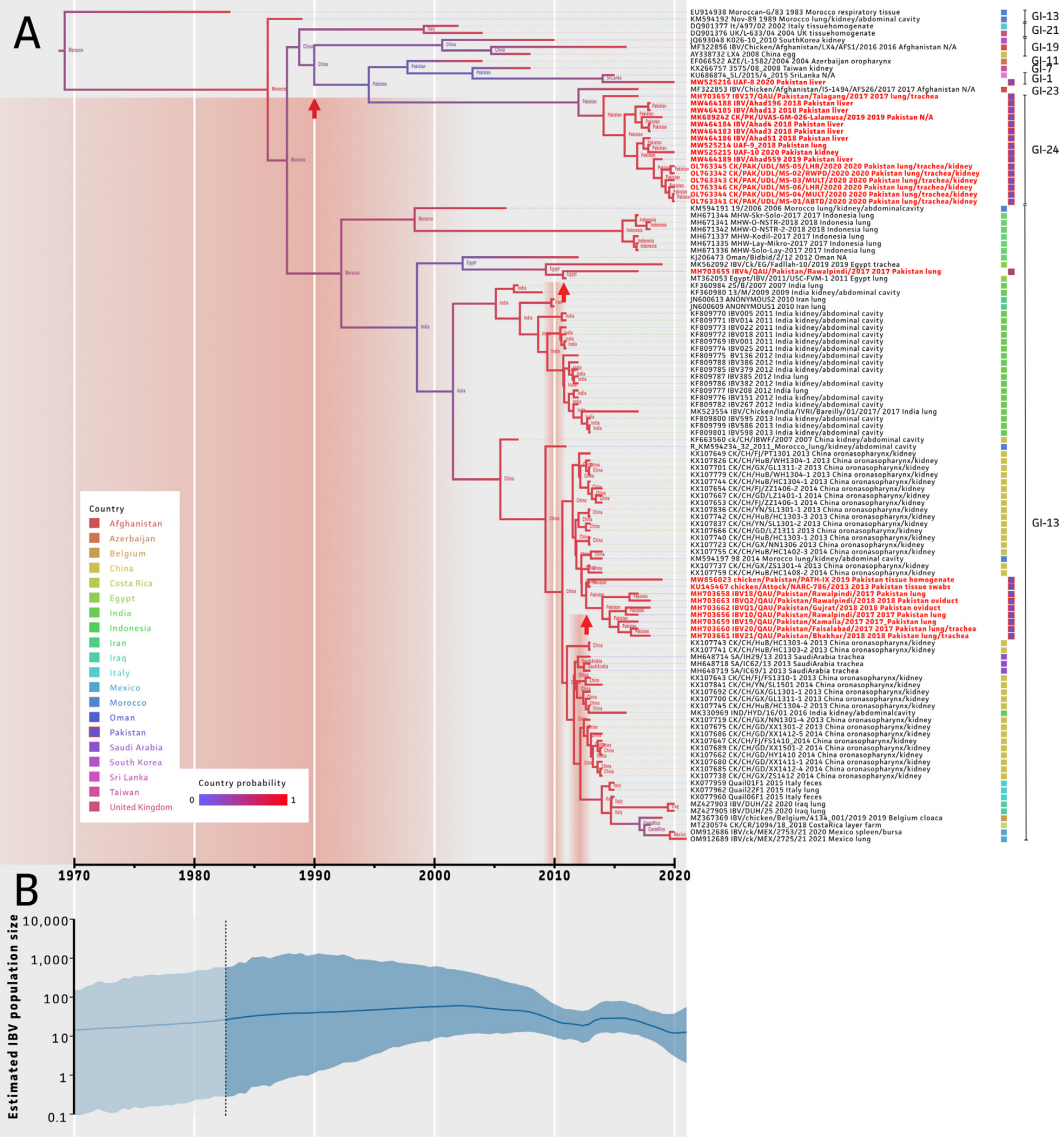
Maximum clade credibility (mcc) tree based on the phylodynamic BEAST analysis. (A) The mcc tree highlights evolutionary relatedness of IBV sequences in relation to their origin. Branches are colored based on the node probabilities in which red and blue indicates high and low probabilities, respectively. Nodes were annotated with the highest likely country at the most common ancestor of 2 branches. Tree tips were annotated with colored boxes based on the country of isolation. Isolation sources are indicated in the isolate names. Genotyping was concluded from Figure 2. (B) Estimated IBV population size based on the Skygrid reconstruction from the BEAST analysis, gray zone with dotted line represents the upper limit of the 95% HPD interval of the estimated date of the root of the tree.

Within the current dataset, the evolutionary rate of the 393 bp within the S1 subunit was estimated to be 5.03 × 10^−3^ nucleotide substitutions per site per year with a 95% highest posterior density (**HPD**) interval of [1.78 × 10^−3^, 9.31 × 10^−3^]. Also, the most recent common ancestor (**mrca**) was estimated at 1962 [1920, 1983]. As shown in Figure 3A, Pakistani sequences clustered in 2 separate clades within the maximum clade credibility (**mcc**) tree. By inferring geographical locations on the tree nodes, putative introductions into Pakistan could be traced. This highlighted at least 3 introduction events that took place in Pakistan (red upward arrows in Figure 3A). A first putative introduction event occurred between 1859 and 2003 (95% HPD interval) but the exact source could only be unraveled with lower confidence as indicated by the blue-colored branches/nodes in the mcc tree. Highest probabilities were obtained for China (0.44) and Pakistan (0.24). Even though less confidence is given to the first introduction event, our data suggested a potential transfer of IBV from Pakistan to Afghanistan (GI-23), Sri Lanka (GI-1), Taiwan (GI-7), and Azerbaijan (GI-11). For the potential introduction into Afghanistan from Pakistan, a probability of 0.60 was obtained as compared to introduction from Afghanistan (0.15) or China (0.14). A second and third introduction is represented by 2 independent GI-13 introductions. A first G-13 introduction is likely to have an origin from Egypt (0.90 probability) and occurred between 2009 and 2011. The second GI-13 introduction was shown to have likely occurred from China to Pakistan between 2011 and 2013 (0.95 probability). An introduction from Morocco or India were considered less likely as these showed low probability measures of 0.02 and 0.03, respectively. As shown in Figure 3B, our dataset allowed to estimate the actual IBV population size over time with a focus on Pakistan and its neighboring countries. A reduction in the IBV population diversity was observed from around 2010s, which had an apparent link with the first introduction of the G-13 strain in Pakistan in that same period. Then, from around 2013 up to 2015, the estimated IBV population size increased again, followed by a slow reduction up to recent days.

### Protein Diversity of Pakistani IBV Strains

**Figure 4:**
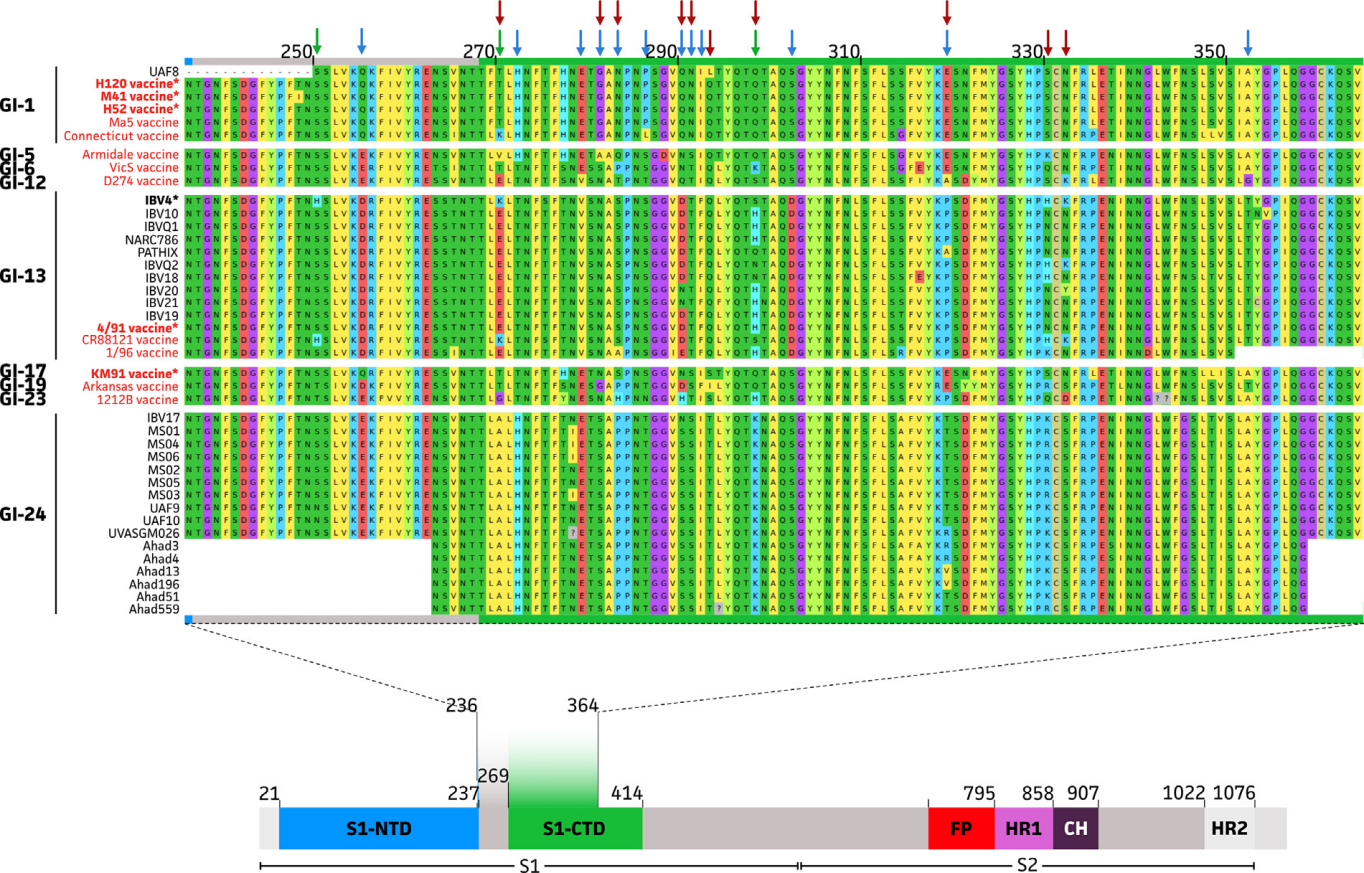
Multiple protein sequence alignment of the partial S1 sequence. The used IBV S1 subunit is highlighted in the complete Spike context as adapted from Shang et al. (2018). Indicated locations represent amino acid locations within the spike protein with a highlight on the used amino acids range between 236 and 364 for all available Pakistani sequences. Also, vaccine strains commonly used in Pakistan (GI-1: H120 (KF188436), M41 (X04722), and H52 (AF352315); GI-13: 4/91 (KF377577); GI-17: KM91 (FJ807946)) and other main vaccine strains as described by Guzmán and Héctor (2020) (Guzmán and Hidalgo, 2020) were included (GI-1: Ma5 (AY561713) and Connecticut (KF696629); GI-5: Armidale (KU556805); GI-6: VicS (KF460437); GI-12: D274 (MH021175); GI-13: CR88121 (JN542567) and 1/96 (MK680010); GI-19: Arkansas (GQ504721); GI-23: 1212B (KU979007)). For vaccine strains B48, 249G, and PL84084 no publicly available sequence could be found. Amino acids were colored per standard color-code as available in MEGA 11. Arrows indicate unique amino acid alterations within the GI-13 IBV4 strain (MH703655, indicated in bold with an asterisk (*)), common GI-13 amino acid alterations, and “high variability” amino acid positions in green, blue, and red, respectively. A question mark (?) indicates nucleotide sequences were lacking in the original sequence file.

The independent GI-13 lineage introductions of IBV into Pakistan were further confirmed by looking at the amino acid differences within the sequenced data. This showed unique amino acid changes to be present in the protein of the isolate with putative origin from Egypt (IBV4; MH703655). Unique alternations were observed as S250H, T270K, and Q298S (as compared to the GI-1 UAF8 Pakistan strain) (Figure 4 green arrows). Still other amino acid changes were observed, which were also identified in other strains belonging to the GI-13 lineage. These included the Q255D, H272T, E279V, G281N, N283S, P286S, Q290D, N291T, I292F, L293Q, S302D, E319P, and A352T (as compared to the GI-1 UAF8 Pakistan strain) (Figure 4 blue arrows). Moreover, some positions seemed to be highly impacted by evolutionary change as most amino acid changes happened at these positions when comparing GI-1, GI-13, and GI-24 protein sequences. Among these were the T270 (T270E and T270A in GI-13 and GI-24 lineages, respectively), G281 (G281N and G281S in GI-13 and GI-24 lineages, respectively), N283 (N283S and N283P in GI-13 and GI-24 lineages, respectively), Q290 (Q55D/N and Q55N/S in GI-13 and GI-24 lineages, respectively), N291 (N291T and N291S in GI-13 and GI-24 lineages, respectively), L293 (L293Q and L293T in GI-13 and GI-24 lineages, respectively), Q298 (Q298S/H/Q/N and Q298K in GI-13 and GI-24 lineages, respectively), E319 (E319P/A and E319T/R/V in GI-13 and GI-24 lineages, respectively), S330 (S330H/N and S330K/R in GI-13 and GI-24 lineages, respectively), and N332 (N332K/N/Y and N332S in GI-13 and GI-24 lineages, respectively) (Figure 4 red arrows). When comparing the Pakistani protein sequences with the used vaccine strains, the GI-1 and GI-13 strains are showing high sequence homology with the used (Figure 4 vaccines with *) vaccines. Only for the GI-24, no closely related vaccine strain seems to be used in Pakistan. Also, when considering other major vaccine strain, none of them showed closer sequence homologies with the GI-24 strains. Interestingly, the closest matching vaccine strain of these GI-24 strains was the 1212B vaccine (GI-23 lineage). Still, significant amino acid changes within the S1 subunit were observed. These were highlighted by 16 amino acid changes, of which 10 were considered significant changes to amino acids belonging to a different group (G270A, T272H, Y277I, H283P, H290N/S, H298K, S314A, P319T/R/V, Q330K/R, and D332S). Also, some strains (*n* = 4) showed an N278I mutation. This indicates that IBV is rapidly undergoing genetic alterations in Pakistan which is resulting in the spread of unique genotypic variants like GI-24 which was not reported before.

## DISCUSSION

IB is a an endemic disease in Pakistan causing enormous economic losses in terms of mortality and decreased production in layer birds (Rafique et al., 2018a). Most studies in Pakistan are based on the seroprevalence and molecular detection of circulating IBV strains, with D-274, D-1466, 4-91, and M-41 IBV strains detected and ELISA-based prevalence rates of up to 98% in layer birds (Ahmed et al., 2007; Fayyaz et al., 2023). Data on genotyping of Pakistani isolates are scarce with only a few studies like that one of Rafique et al., in which they have genotypically characterized 1 isolate (Rafique et al., 2018a). Similarly, data in relation to geographic distribution and sampling origin of IBV isolates are limited. This study was aimed at phylogeographic characterization of all available Pakistani isolate sequences, including 9 new field isolates, based on the partial S1 sequence (393 bp). We found that all available Pakistani isolates belong to a single genotype (GI), with strains belonging to the GI-1 (*n* = 1), GI-13 (*n* = 10), and GI-24 (*n* = 16) lineages. Key difference between these lineages was recently highlighted by a study on cleavage recognition motifs of S1 gene, with GI-1 and GI-24 having Arg-Arg-Phe-Arg-Arg (RRFRR) while GI-13 having Arg-Arg-Ser-Arg-Arg (RRSRR) motifs (Huang et al., 2020).

Among these lineages, GI-13 is a widely circulating genotype worldwide including many Asian and Middle-Eastern countries (Valastro et al., 2016; Bali et al., 2021). It includes both virulent and vaccine strains, previously assigned to the 793/B type (also known as 4/91 and CR88) (Valastro et al., 2016). One study characterized a Pakistani isolate (KU145467) in this lineage (Rafique et al., 2018a). In our study, 9 more isolates were classified to this lineage. All samples originated from respiratory tract or oviduct. A recent study observed the pathogenesis 4/91 IBV in laying hens. Although no pathological signs in reproductive tract were observed, there was a significant drop in egg production and quality (Najimudeen et al., 2021), suggesting that GI-13 lineage strains have affinity for the reproductive tract as well. The GI-1 lineage represents the most commonly used Massachusetts (**Mass**) vaccine strain and is also widely present in IBV endemic countries (Houta et al., 2021). One isolate from our study (MW525216) belonged to this lineage and originated from the liver. Studies have shown that GI-I lineage strains, particularly M41 show vast tissue tropism including respiratory tract, spleen, liver, gastrointestinal tract and kidney (Abdel-Moneim et al., 2009). Interestingly, the GI-24 lineage is peculiar in this regard as these strains show significant amino acid differences with any of the commonly used vaccine strains (Figure 4). This lineage was reported to be unique to India based on 24 isolates collected between 1998 and 2013 (Valastro et al., 2016; Raja et al., 2020). To the authors’ knowledge, only 1 Chinese isolate was reported to be classified to this GI-24 lineage (Ji et al., 2020). In our study, 16 out of 27 Pakistani sequences belonged to this lineage, originating from respiratory tract, liver, or kidney. Raja et al. also isolated GI-24 isolates from kidneys (Raja et al., 2020), showing the nephropathogenic nature of this lineage.

The IBV genome undergoes significant mutations due to limited proof-reading ability of viral polymerase with an average rate of 1.2 × 10^−3^ synonymous substitutions/site/year (Holmes, 2009; Moharam et al., 2020). In our study, this rate in our 393 bp target was estimated to be within that same order of magnitude, 5.03 × 10^−3^ nucleotide substitutions per site per year with a 95% HPD interval of [1.78 × 10^−3^, 9.31 × 10^−3^]. The S1 protein is of utmost importance as mutations within this part lead to emergence of new strains (Saadat et al., 2017). Three hypervariable regions (HVR 1, aa 38–67; HVR 2, aa 91–141; and HVR-3, aa 274–387) in this protein are hotspots of amino acid substitutions (Moharam et al., 2020). In this study, the target protein sequence falls within the HVR-3 (aa 236–364, Figure 4). A study in Costa Rica found 3 amino acid substitutions between positions 338 and 350 in the GA13-CR cluster within the GI-17 lineage resulting in a frameshift mutation (Villalobos-Agüero et al., 2022). In our study, IBV4 of GI-13 lineage also showed peculiar substitutions in a nearby region at amino acid positions 330 to 338 which lead to similar mutations (Figure 4). Another Egyptian isolate with a GI-13 parent lineage showed a peculiar substitution at A281P (Rohaim et al., 2019). In GI-1 lineage, a single Pakistani isolate was characterized with key substitutions at Q294L as compared to vaccine strains H-120, M-41, and H-52. A recent study in Egypt was in line with these results suggesting a lacked glycosylation site at position 284 of an IBV variant when compared to H-120 (Ameen et al., 2023). Most Pakistani strains belonged to the GI-24 lineage, which is not described before from the country. Since there is no known vaccine strain for this lineage, no conclusions could be drawn here. Still, key mutations within this lineage when compared to commonly used vaccine strains in Pakistan indicate that further exploration of HVR3 with respect to GI-24 in particular and GI-1 and GI-13 in general will provide vital information about heterogeneity of circulating IBV strains in Pakistan.

Based on the phylogeographic analysis in the BEAST software, estimations on the tree node origins could be made to trace 3 possible introduction events of IBV into Pakistan, 2 of which showed a very high probability (Egypt and China). First introduction event to and from Pakistan has low probability and could be due to the geography of the area. A selection of isolates with sufficient and accurate metadata (location, time of collection, and tissue of isolation) was performed. Inclusion of a broader range of isolates from more (neighboring) countries and time points might improve the estimations here. Still, more sequencing efforts are thought to be required in South Asia to facilitate this type of analyses. Migratory birds may play a possible role in this over a long period of time (Wille and Holmes, 2020), traveling from colder regions of Afghanistan and China to warmer regions of Pakistan during winter and vice versa. A second introduction event happened from Egypt to Pakistan between 2009 and 2011 (0.90 probability). Although, during this time frame, there are no reports of any poultry import in Pakistan directly from Egypt, still Egypt is a big exporter of poultry products to Saudi Arabia and United Arab Emirates (Woertz and Keulertz, 2015). During 2008 to 2011, Pakistan imported significant poultry products from the gulf countries (OEC; OEC, 2023), hinting toward a possible route of first introduction of the GI-13 lineage (Figure 3A). This is further supported by 98.56 to 99.59% similarity of 4 Egyptian isolates to the Pakistani strain of GI-13 lineage (Ghorbiani et al., 2020). A third introduction (GI-13 lineage) event occurred through China between 2011 and 2013 (0.95 probability). Since 2012, Pakistan started importing frozen poultry products from China (Ghorbiani et al., 2020), which is a potential source of this new GI-13 lineage introduction into Pakistan. With the establishment of China-Pakistan Economic Corridor (**CPEC**) in 2015, more introduction events from China can be expected. This enhances the importance of phylogeographical studies as shown here, which are based on accurate sequencing data and associated metadata. More studies are needed on the genetic characterization of circulating field isolates in Pakistan to get a more accurate picture on the diversity and phylogeographic relationship of IBV strains.

This analysis also allowed to estimate the actual IBV population size over time, which highlighted a reduction of the population, which coincided with the 2 GI-13 introduction events in Pakistan. The GI-13 strain likely became the predominant lineage in Pakistan in this period as it is known to be widely circulating across the world (Valastro et al., 2016; Bali et al., 2021). After this, increased biosecurity (e.g., management and change in vaccination strategies) are thought to have resulted in a reduction of the further spread of the GI-13 lineage in Pakistan. Even though actual data on these events are scarce, an increase of the IBV population was observed around 2014 again. The use of this GI-13 vaccine (4/91), might have resulted in the selection for novel and/or other circulating lineages (e.g., GI-24) to cause vaccination breakthrough infections as previously reported for SARS-CoV-2 (Keyel et al., 2022). This again suggests that the GI-24 is the prevalent circuiting field genotype, and currently no vaccination is targeted against this lineage which may result in increase of IBV population in coming years. This also necessitates the use of GI-24 based vaccines in Pakistan and India, a strategy which was adopted to a significant effect by a study that used GI-23 based wild-type IBV (Eg/1212B/2012) vaccination to control the newly emerging lineage in the Middle-East (Sultan et al., 2019).

In our study, IBV 17 of GI-24 lineage showed alterations as Q255E, H277T, S282P, E319T, P319T, S330K, and L335P (compared to KM91 vaccine strain of GI-23, which is currently being used in Pakistan by ICI Pakistan Ltd.). These differences can result in certain mutations which allow GI-24 lineage strains to evade the vaccines targeted against GI-23 lineage. Other amino acid differences within the sequenced data also highlighted the key changes in the selected S1 protein changes. This information suggests that GI-24 is taking over as dominant lineage in Pakistani field IBV isolates over the last years (2017–2020). As there are no vaccination strains currently being used in this lineage, it can allow further spread. It also opens doors toward manufacturing of live attenuated and killed vaccines based on this lineage in the Indian subcontinent.

Even though our study focused on the S1, alterations in the S2 have also been linked with increased neurotropism in QX genotype (Cheng et al., 2019). Also, IBV nonstructural protein (**nsp**) 2, 3, and 16 coding regions also showed high variability (Thor et al., 2011). Other studies have speculated that changes in the nsp6 and nsp9 can alter the pathogenicity of IBV (Quinteros et al., 2015). This suggests that, despite S1 being the most explored region of IBV genome, other parts of its genome are also prone to mutations, altering the pathogenicity and replication of virus, and should be explored when studying genetic correlation and genotyping. Thus, supporting the need for more sequencing data on complete-coding genomes of IBV strains across the world.

## CONCLUSIONS

This study phylogeographically characterized the status of IBV diversity with currently available IBV isolates from Pakistan. All Pakistani isolates belonged to genotype I (GI), with the GI-24 lineage being of particular interest as no closely related vaccine is currently available in Pakistan and in the world. Our study also highlighted a minimum of 3 independent IBV introduction events into Pakistan over the last 5 decades and estimated the circulating IBV population in Pakistan and its neighbors over this period. Amino acid changes in the strains also highlighted the differences between genotypic lineages, showing that the IBV isolates acquired key mutations over the years resulting in spread of novel genotypes not reported before. The results highlight the importance of IBV genotyping and phylogeographic analyses to trace the spread and introductions of IBV lineages, which will also aid in the design of new vaccines and setting up eradication campaigns.

## Appendix Supplementary materials

Image, application 1

Image, application 2

## Footnotes

Supplementary material associated with this article can be found in the online version at doi:10.1016/j.psj.2023.103236.
