# Supplementary material for: Genotyping and phylogeography of infectious bronchitis virus isolates from Pakistan show unique linkage to GI-24 lineage
Source: Poult Sci. 2023 Oct 24;103(1):103236. doi: 10.1016/j.psj.2023.103236 (PMC10685022; doi:10.1016/j.psj.2023.103236)
Supplement: Supplementary file 1 [file mmc1.pdf]

Table S1: An overview of sequences by countries and sample types

Parameter	Number	Description
New IBV isolates	9	MH703655 - MH703663
Other Pakistani isolates	18	2013-2020<br>Lung, trachea, kidney, liver<br>1 unknown
Other Asian isolates	77	China: 35<br>India: 21<br>Indonesia: 6<br>Saudi Arabia: 3<br>Afghanistan: 2 (tissue unknown)<br>Sri Lanka: 2 (tissue unknown)<br>Iran: 2<br>Iraq: 2<br>Oman: 1 (tissue unknown)<br>Azerbaijan: 1<br>South Korea: 1<br>Taiwan: 1
African isolates	7	Morocco: 5<br>Egypt: 2
European isolates	6	Italy: 4<br>UK: 1<br>Belgium: 1
Central American isolates	3	Mexico: 2<br>Costa Rica: 1
Total	120	Time Period: 1983-2021<br>Countries: 18<br>Isolation sources: 13<br>1. Lung<br>2. Trachea<br>3. Oviduct<br>4. Kidney<br>5. Liver<br>6. Abdominal cavity<br>7. Cloaca<br>8. Oronasopharynx<br>9. Spleen/Bursa<br>10. Feces<br>11. Egg<br>12. Tissue homogenate<br>13. Tissue swabs
