# Supplementary material for: Genotyping and phylogeography of infectious bronchitis virus isolates from Pakistan show unique linkage to GI-24 lineage
Source: Poult Sci. 2023 Oct 24;103(1):103236. doi: 10.1016/j.psj.2023.103236 (PMC10685022; doi:10.1016/j.psj.2023.103236)
Supplement: Supplementary file 2 [file mmc2.pdf]

Table S2: List of Sequences (accession numbers) used in this study:

S. No.	Accession numbers
1.	MH703655
2.	MH703656
3.	MH703657
4.	MH703658
5.	MH703659
6.	MH703660
7.	MH703661
8.	MH703662
9.	MH703663
10.	OL763341
11.	OL763342
12.	OL763343
13.	OL763344
14.	OL763345
15.	OL763346
16.	MW856023
17.	KU145467
18.	MW464183
19.	MW464184
20.	MW464185
21.	MW464186
22.	MW464188
23.	MW464189
24.	MW525214
25.	MW525215
26.	MW525216
27.	MK689242
28.	MZ427903
29.	MZ427905
30.	MT362053
31.	MK562092
32.	MK523554
33.	KX077962
34.	KF809777
35.	KF809787
36.	KF360984
37.	JN600609
38.	JN600613
39.	MH671335
40.	MH671336
41.	MH671337
42.	MH671341
43.	MH671342
44.	MH671344
45.	EU914938
46.	KM594191
47.	KM594192

48.	KM594197
49.	KM594234
50.	MZ367369
51.	MK330969
52.	KF809769
53.	KF809770
54.	KF809771
55.	KF809772
56.	KF809773
57.	KF809774
58.	KF809775
59.	KF809776
60.	KF809782
61.	KF809785
62.	KF809786
63.	KF809788
64.	KF809799
65.	KF809800
66.	KF809801
67.	KF360980
68.	KF663560
69.	KX077959
70.	KX077960
71.	DQ901376
72.	DQ901377
73.	JQ693048
74.	KX266757
75.	KX107643
76.	KX107647
77.	KX107649
78.	KX107653
79.	KX107654
80.	KX107662
81.	KX107666
82.	KX107667
83.	KX107675
84.	KX107680
85.	KX107685
86.	KX107686
87.	KX107689
88.	KX107692
89.	KX107700
90.	KX107701
91.	KX107719
92.	KX107723
93.	KX107737
94.	KX107738
95.	KX107740
96.	KX107741
97.	KX107742

98.	KX107743
99.	KX107744
100.	KX107745
101.	KX107755
102.	KX107759
103.	KX107779
104.	KX107826
105.	KX107836
106.	KX107837
107.	KX107841
108.	AY338732
109.	MH648714
110.	MH648718
111.	MH648719
112.	KU686874
113.	KU686875
114.	MF322853
115.	MF322856
116.	KJ206473
117.	EF066522
118.	MT230574
119.	OM912686
120.	OM912689
